# Strain‐Driven Selective Stabilization of Metastable TiO_2_ Phases

**DOI:** 10.1002/smll.202505427

**Published:** 2025-09-18

**Authors:** Jihoon Jeon, Myungsu Jang, Gwang Min Park, Minseok Kim, Jongseo Kim, Seungwan Ye, Yongjoo Park, Seung‐Hyub Baek, Jun‐Yun Kang, Seong Keun Kim

**Affiliations:** ^1^ KU‐KIST Graduate School of Converging Science and Technology Korea University Seoul 02841 Republic of Korea; ^2^ Electronic and Hybrid Materials Research Center Korea Institute of Science and Technology Seoul 02792 Republic of Korea; ^3^ Advanced Research Development Team SK Trichem Sejong 30068 Republic of Korea; ^4^ Extreme Materials Research Institute Korea Institute of Materials Science Changwon 51508 Republic of Korea

**Keywords:** atomic layer deposition, metastable phase, rutile, TiO_2_‐II

## Abstract

Stabilizing metastable TiO_2_ phases in thin films remains a significant challenge. This paper demonstrates a strain‐driven approach for selectively stabilizing the metastable phases of orthorhombic TiO_2_‐II and rutile using (111)‐oriented face‐centered cubic (FCC) metal substrates via low‐temperature atomic layer deposition. Epitaxial FCC metal substrates, including Ir and Pt, exhibit a strong preferential (111) orientation and promote the formation of TiO_2_‐II with a preferential (200) orientation through favorable lattice matching. In contrast, TiO_2_ grown on (111)‐textured polycrystalline FCC metals crystallizes into rutile with a preferential (110) orientation despite identical growth conditions, which is attributed to strain relaxation arising from the random in‐plane orientations of FCC metals. Compared to the stable anatase phase, TiO_2_‐II films exhibit higher density (4.45–4.51 g cm^−3^), higher refractive indices, and higher dielectric constants (≈75–77). These findings reveal that in‐plane strain and lattice matching can be strategically utilized to engineer metastable TiO_2_ phases, offering a new approach for the phase‐selective growth of functional oxide films at low temperatures.

## Graphical abstract

This work presents a strain‐guided approach for selectively stabilizing metastable TiO_2_ polymorphs using FCC metal substrates. Epitaxial Pt and Ir promote orthorhombic TiO_2_‐II growth through lattice matching and strain preservation, while polycrystalline substrates lead to rutile formation due to in‐plane strain relaxation. The results offer a route for controlled phase engineering in oxide thin films.

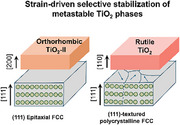

## 1 Introduction

TiO_2_ is well known for its diverse polymorphs, including the extensively studied anatase, rutile, and brookite phases, and several high‐pressure phases. Each polymorph has a different coordination of Ti and O atoms and different bond lengths, leading to distinct chemical, optical, and electrical properties. For instance, anatase exhibits excellent photocatalytic activity under UV irradiation, which is attributed to its high charge‐carrier mobility.^[^ ^1^ ^]^ Rutile, which has poor photocatalytic activity owing to its low UV absorption, has a high refractive index and high dielectric constant (170 along the c‐axis and 86 along the a‐axis).^[^ ^2^ ^]^ Its high dielectric constant makes it particularly interesting as a dielectric layer for dynamic random‐access memory (DRAM) capacitors.^[^ ^3^, ^4^, ^5^, ^6^, ^7^, ^8^ ^]^ Among the less‐studied polymorphs, orthorhombic TiO_2_‐II (α‐PbO_2_‐type structure; space group Pbcn; lattice constant: a = 0.454 nm, b = 0.551 nm, c = 0.491 nm)^[^ ^9^ ^]^ is known for its exceptional mechanical strength due to its high density and bulk modulus.^[^ ^10^, ^11^ ^]^ Moreover, first‐principles calculations predicted a high dielectric constant for TiO_2_‐II (≈74 along the a‐axis),^[^ ^11^ ^]^ although this value has not yet been experimentally verified. The diverse properties of TiO_2_ polymorphs, which are determined by their crystal structures, enable a wide range of applications. This background highlights the need for strategies to selectively stabilize specific crystal phases, even if the resulting structures are metastable.

**Figure 1:**
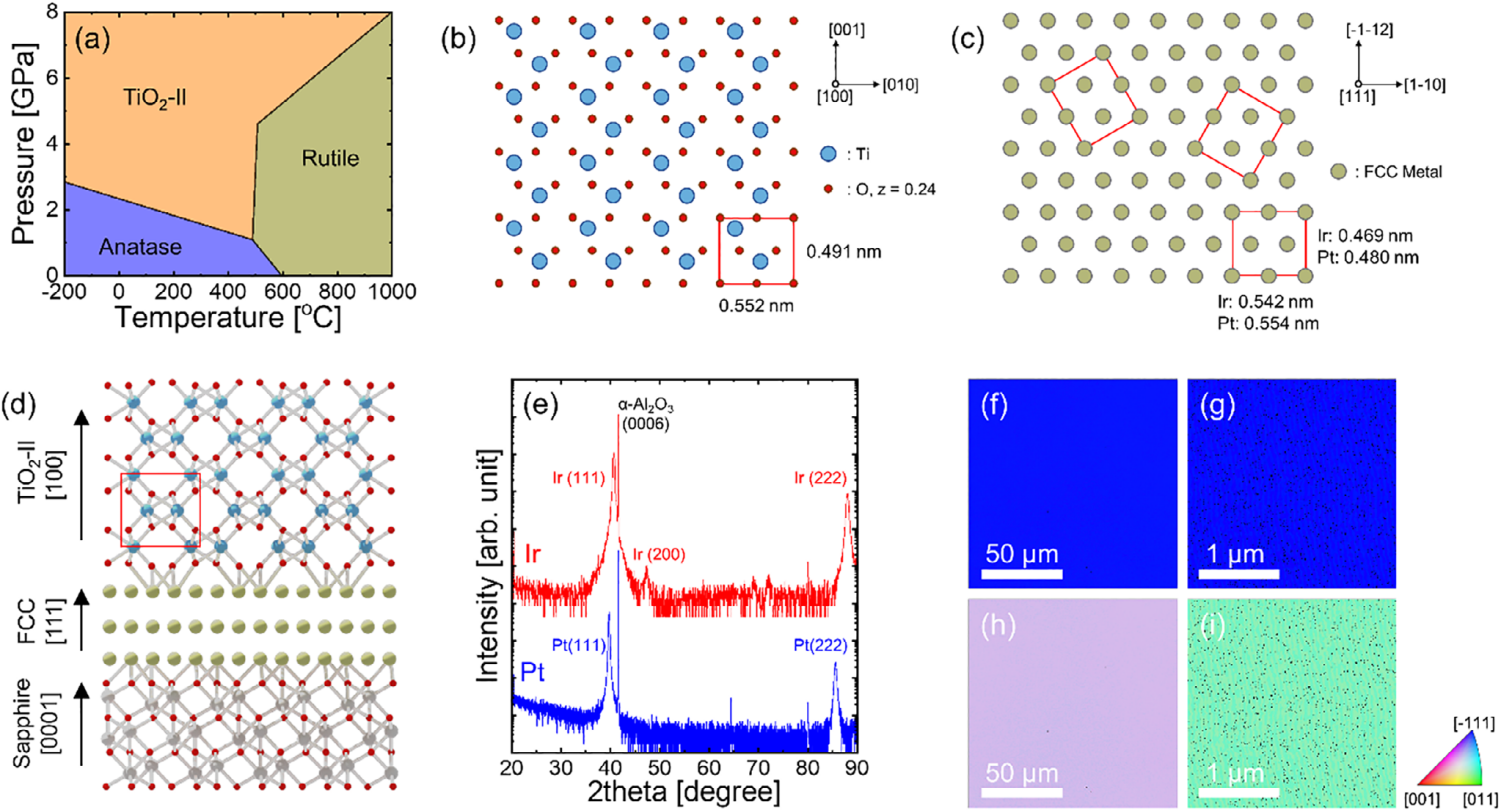
a) Phase diagram of several TiO_2_ phases. Schematics of the atomic arrangements of the (b) (100) plane of TiO_2_‐II and c) (111) plane of FCC metals, including Ir and Pt. d) Schematic of the orientation‐locked heteroepitaxy of the orthorhombic TiO_2_‐II on a FCC (111) layer on a c‐cut sapphire substrate. e) θ–2θ XRD patterns of Ir and Pt films grown on c‐cut sapphire. EBSD f, g) out‐of‐plane and h, i) in‐plane orientation maps of (f, h) Ir and (g, i) Pt films grown on c‐cut sapphire.

Atomic layer deposition (ALD) is a thin‐film growth technique based on the self‐limiting adsorption of reactants on a reaction surface. ALD is a low‐temperature processing method that achieves excellent conformality of complex‐shaped structures and high‐quality film deposition. Extensive studies have reported ALD TiO_2_ films, which were mostly deposited at temperatures below ≈350 °C owing to the low thermal stability of commonly used metal–organic Ti precursors.^[^ ^12^, ^13^ ^]^ As shown in the phase diagram in **Figure** **1**a, anatase is thermodynamically stable at the low temperatures typically used for ALD, whereas stabilizing the rutile phase requires high temperatures, and the TiO_2_‐II phase requires high pressure.^[^ ^14^, ^15^ ^]^ Consequently, conventional ALD predominantly yields anatase‐phase TiO_2_ films.^[^ ^16^, ^17^, ^18^, ^19^, ^20^ ^]^

Several studies have demonstrated the stabilization of metastable TiO_2_ phases via ALD. One approach involves using a substrate that is structurally compatible with rutile. A bottom layer of rutile‐structured materials, such as RuO_2_, IrO_2_, and SnO_2_, as well as MoO_2_ with a distorted rutile structure, has been applied to facilitate rutile TiO_2_ growth at temperatures below 300 °C.^[^ ^3^, ^21^, ^22^, ^23^, ^24^, ^25^, ^26^, ^27^, ^28^, ^29^, ^30^, ^31^, ^32^ ^]^ In addition, orthorhombic TiO_2_‐II films were prepared by ALD using TiCl_4_ as an inorganic precursor.^[^ ^33^, ^34^, ^35^ ^]^ However, this approach required temperatures above 400 °C, and the mechanism responsible for stabilizing the orthorhombic phase was not clarified.

In this paper, we report the selective stabilization of metastable TiO_2_ phases using face‐centered cubic (FCC) metal films as substrates. The (111) plane of FCC metals is structurally compatible with both orthorhombic TiO_2_‐II and rutile phases. We found that the selective stabilization of these TiO_2_ phases was determined by the structure of the FCC metal layers, offering a new strategy for phase‐controlled oxide‐film engineering.

## 2 Results and Discussion

First, we examined the structural compatibility between TiO_2_‐II and FCC metals to confirm the possibility of stabilizing the TiO_2_‐II phase. Figure 1b,c show the atomic arrangements of the (100) plane of TiO_2_‐II and the (111) plane of the FCC metals, including Ir and Pt, respectively. Both schematics show rectangular in‐plane unit cells for each plane. The edge parameters of these unit cells were calculated using the lattice constants of TiO_2_‐II and the FCC metals listed in **Table** **1**. The lattice mismatches along the [001] direction of TiO_2_‐II are −2.25% for Pt and −4.66% for Ir, while those along the [010] direction are 0.43% for Pt and −1.90% for Ir; these values indicate small mismatches in both directions. This favorable in‐plane orientational relationship between TiO_2_‐II and FCC metals highlights the possibility of using (111)‐orientated FCC metal films to promote the stabilization of metastable TiO_2_‐II.

**Table 1:** Lattice parameters of FCC metals and edge parameters along the [1‐10] and [‐1‐12] directions.

FCC metal	Lattice parameter [nm]	FCC [1‐10] [nm]	FCC [‐1‐12] [nm]
Ir	0.383	0.542	0.469
Pt	0.392	0.554	0.480

To verify this hypothesis, we deposited FCC metal films of Ir and Pt on single‐crystal c‐cut sapphire substrates, followed by the growth of TiO_2_ films via ALD. Figure 1d schematically illustrates the orientation‐locked heteroepitaxy of orthorhombic TiO_2_‐II on the FCC (111) coating on a c‐cut sapphire surface. The (0001) plane of sapphire exhibits structural compatibility with the (111) plane of FCC metals, such as Ir and Pt (Figure S1, Supporting Information), further enhancing the preferential (111) orientation of the Ir and Pt films.

Figure 1e shows the θ–2θ X‐ray diffraction (XRD) patterns of Ir and Pt films grown on c‐cut sapphire. Both films exhibit strong (111) Bragg peaks. In addition, the full width at half maximum (FWHM) of the rocking curves for the (111) peaks was as low as 1.17° for Ir and 0.81° for Pt (Figure S2, Supporting Information), confirming a strong (111) preferential orientation. Although the Ir film also showed minor peaks, such as the (200) reflection, their intensities were significantly weaker than those for the Pt film. Electron backscatter diffraction (EBSD) maps of the out‐of‐plane orientation in Figure 1f,g further verify that both the Ir and Pt films show a preferential (111) orientation across the entire analyzed region. In addition, the in‐plane orientation maps in Figure 1h,i show that both films have a uniform crystallographic orientation. Furthermore, XRD φ scans of the Ir/α‐Al_2_O_3_ and Pt/α‐Al_2_O_3_ (Figure S3, Supporting Information) reveal that the (113) peaks of Ir and Pt are aligned with the (113) peaks of α‐Al_2_O_3_ at the same φ angles. These results indicate that the FCC metal films were grown epitaxially on c‐cut sapphire, consistent with the orientation‐locked epitaxy illustrated in Figure 1d.

**Figure 2:**
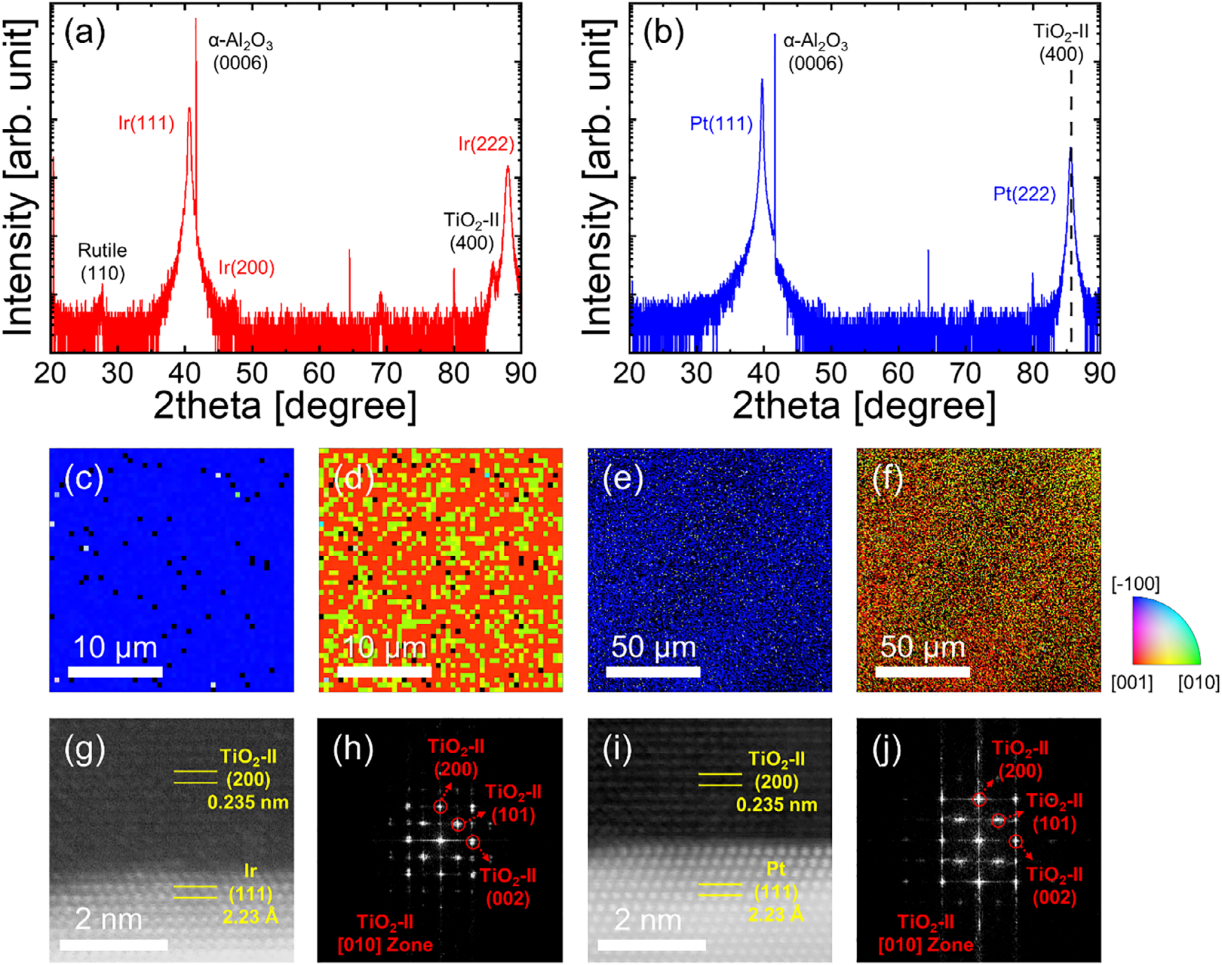
θ–2θ XRD patterns of 20 nm‐thick TiO_2_ films grown on (a) Ir/c‐cut sapphire and b) Pt/c‐cut sapphire. c, e) Out‐of‐plane and d, f) in‐plane orientation EBSD maps of TiO_2_ films grown on (c, d) Ir/c‐cut sapphire and (e, f) Pt/c‐cut sapphire. g, i) STEM images and their corresponding h, j) FFT patterns for TiO_2_ films grown on (g, h) Ir/c‐cut sapphire and (i, j) Pt/c‐cut sapphire.

The TiO_2_ films grown on epitaxial Ir and Pt layers exhibited smooth surfaces without significant interfacial reactions with the metal layers (Figure S4, Supporting Information). TiO_2_ films grown on Si and TiN substrates using an identical ALD process at 330 °C crystallized into the anatase phase (Figure S5, Supporting Information). Hence, we examined the crystalline structure of TiO_2_ films grown on FCC (111) surfaces. **Figure** **2**a,b show the θ–2θ XRD patterns of 20 nm‐thick TiO_2_ films grown on Ir/c‐cut sapphire and Pt/c‐cut sapphire, respectively. The XRD pattern of the TiO_2_ film on Ir exhibits a distinct TiO_2_‐II (400) peak at 85.8°, indicating the effective stabilization of the orthorhombic phase. The TiO_2_‐II (200) peak, typically observed at 39.624°, was not distinguished because it overlapped with the strong Ir (111) peak. Although a rutile (110) peak was also detected, the high intensity of the TiO_2_‐II (400) peak, even at a high 2θ angle, suggests that the film predominantly crystallizes as the TiO_2_‐II phase with an out‐of‐plane a‐axis orientation. Furthermore, the TiO_2_‐II phase is preserved even at a relatively large thickness of ≈70 nm (Figure S6, Supporting Information). The EBSD results shown in Figure 2c also support the stabilization of the TiO_2_‐II phase with a preferential (200) orientation. The in‐plane orientation map in Figure 2d shows multiple distinct in‐plane orientations. This behavior can be explained by the multiple lattice‐matching configurations between the TiO_2_‐II phase and underlying epitaxial Pt and Ir(111) substrates, as shown in Figure 1c. In contrast, the XRD pattern of the TiO_2_ film grown on Pt showed no observable peaks. This is likely due to the overlap of the TiO_2_‐II (200) and (400) peaks with the Pt (111) and (222) peaks, making them difficult to resolve. However, the EBSD results in Figure 2e show that the TiO_2_ film crystallized into TiO_2_‐II with a (200) preferential orientation, which is consistent with the results observed for the TiO_2_/Ir/c‐cut sapphire system. The TiO_2_ films grown on Pt also exhibited several distinct in‐plane orientations (Figure 2f).

Scanning transmission electron microscopy (STEM) was employed to further clarify the TiO_2_ phases. Figure 2g–j show STEM images and corresponding fast Fourier transform (FFT) patterns for (g, h) TiO_2_/Ir and (i, j) TiO_2_/Pt. The STEM images show that the TiO_2_ films grown on the (111)‐oriented Ir and Pt substrates have their a‐axes oriented perpendicular to the substrate surface. The FFT diffraction patterns confirm that orthorhombic TiO_2_‐II films were grown on both substrates. Selected area electron diffraction images (Figure S7, Supporting Information) corroborate this observation, revealing that the TiO_2_ (h00) planes are aligned with the Ir and Pt (111) surfaces, indicating the epitaxial growth of the orthorhombic phase.

**Figure 3:**
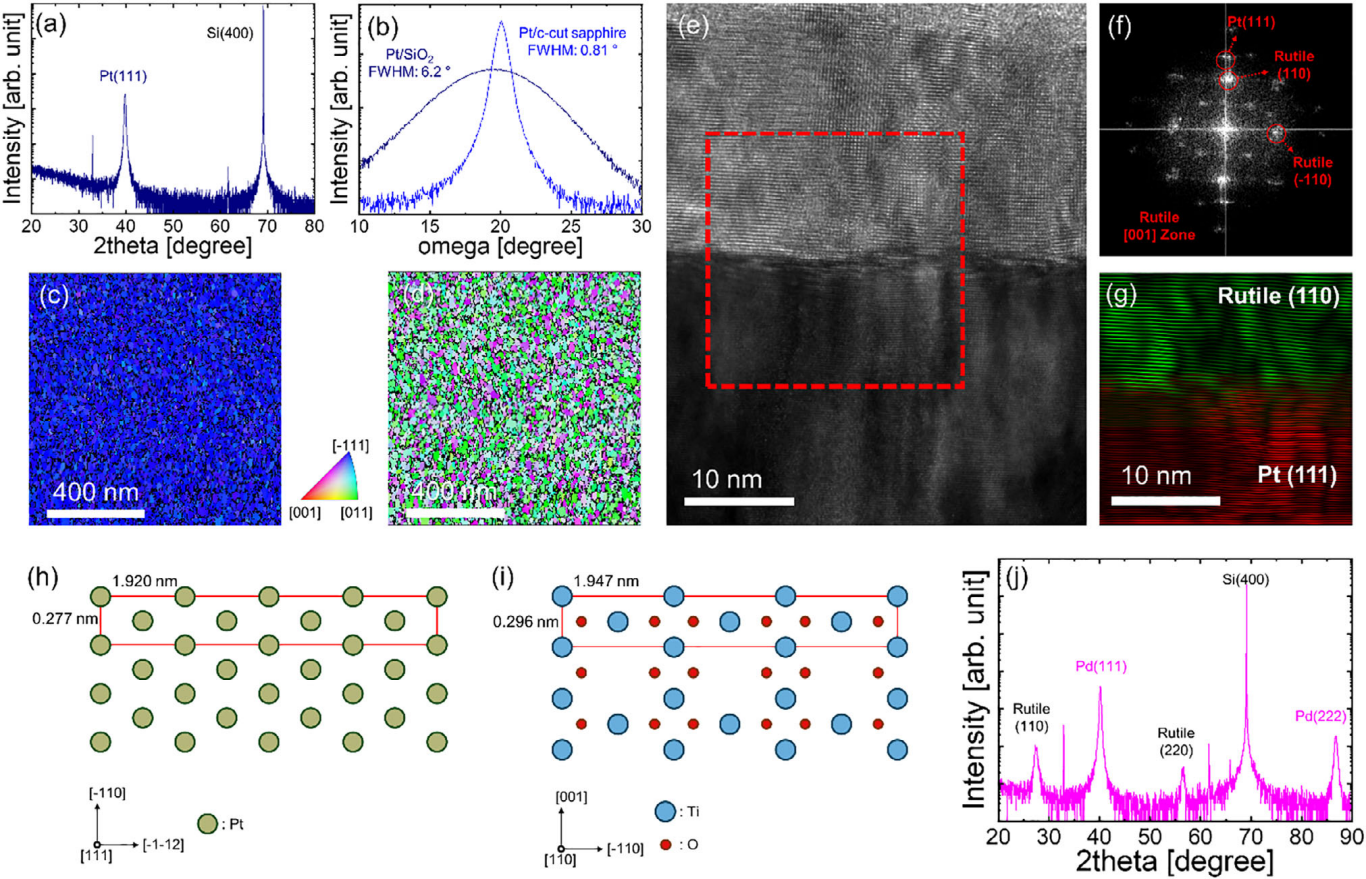
a) θ–2θ XRD pattern of a Pt film grown on an amorphous SiO_2_ substrate by sputtering at room temperature. b) Rocking curves of Pt films grown on amorphous SiO_2_ and c‐cut sapphire. c) Out‐of‐plane and d) in‐plane orientation maps of a Pt film grown on SiO_2_/Si. e) HRTEM image of the TiO_2_ film grown on (111)‐textured polycrystalline Pt layer/SiO_2_. f) FFT pattern obtained from the red‐boxed region in (e). g) Inverse FFT image of rutile (110) and Pt (111) diffraction points. Schematics of the atomic arrangements of (h) the (111) plane of FCC metals, including Ir and Pt, and i) the (110) plane of rutile TiO_2_. (j) θ–2θ XRD patterns of the TiO_2_ film grown on (111)‐textured Pd/amorphous SiO_2_/Si substrates.

The (111) plane is the most closely packed in FCC structures. Thus, FCC metal films tend to grow with a preferred orientation along the [111] direction, even on non‐single‐crystal substrates. Therefore, we further examined the crystal structure of the TiO_2_ films grown on polycrystalline Pt layers with a strong (111) preferential orientation. **Figure** **3**a shows a θ–2θ XRD pattern of the Pt film grown on an amorphous SiO_2_ substrate by sputtering at room temperature. Despite the amorphous substrate, a strong Pt (111) peak is observed. Furthermore, the rocking curve of the Pt (111) reflection in Figure 3b exhibits a broad FWHM of 6.2°, confirming a polycrystalline film. The out‐of‐plane EBSD orientation map in Figure 3c further confirms that most Pt grains have a strong (111) orientation preference. However, the in‐plane orientation map in Figure 3d reveals a wide distribution of crystallographic orientations among very fine grains, which are significantly smaller than those in epitaxially grown Pt films on c‐cut sapphire substrates (Figure 1i).

Figure 3e shows a high‐resolution TEM (HRTEM) image of a TiO_2_ film grown on a (111) surface of a Pt layer using ALD conditions identical to those used for TiO_2_ films on epitaxial FCC metal substrates. Interestingly, the FFT pattern obtained from the red‐boxed region in Figure 3e shows that the TiO_2_ film crystallized into the rutile phase rather than the orthorhombic phase (Figure 3f). Additionally, the diffraction spots corresponding to Pt (111) and rutile (110) planes are closely aligned along the vertical direction, indicating a specific orientational relationship. This was further confirmed by the inverse FFT image in Figure 3g, which clearly shows rutile (110) planes aligned parallel to the Pt (111) surface.

These observations raise two important issues. First, the orthorhombic TiO_2_‐II phase does not form on polycrystalline Pt despite a similar (111) preferential orientation to that of epitaxial Pt substrates. Second, metastable rutile is preferentially stabilized on the polycrystalline Pt (111) surfaces, rather than the more stable anatase phase, which is typically favored on amorphous or polycrystalline substrates.

Although orthorhombic TiO_2_‐II exhibits good lattice matching with the FCC (111) surface, as shown in Figure 1b–d, the polycrystallinity of the Pt layer hinders strain maintenance. As illustrated in Figure 1a, the stabilization of TiO_2_‐II requires the preservation of high in‐plane stress. However, the small randomly oriented grains in the (111)‐textured Pt films disrupt the coherent in‐plane stress across the TiO_2_ film during its growth, resulting in strain relaxation. For these reasons, TiO_2_‐II did not form on the polycrystalline FCC (111) surfaces.

The schematics in Figure 3h,i provide insights into why rutile, as another metastable phase, is preferentially stabilized on (111)‐textured Pt rather than TiO_2_‐II or anatase (the most stable phase). A notable lattice match exists between the Pt (111) surface and the rutile TiO_2_ (110) plane. The lattice spacing is 0.296 nm along the [001] direction of rutile TiO_2_, which closely matches the 0.277 nm spacing of Pt along the in‐plane direction, resulting in a small lattice mismatch of 6.1%. Along the [−110] direction of rutile, the unit cell length is 1.947 nm, which is comparable to the corresponding periodicity of the Pt (111) surface, 1.920 nm. Although not a perfect match, the use of extended periodicities can mitigate mismatch strain. The small lattice mismatches along both in‐plane directions indicate a favorable domain matching epitaxial relationship, in which the rutile TiO_2_ (110) plane aligns well with the Pt (111) plane, promoting the growth of the rutile phase despite the structural differences between the two materials.

To further verify the domain epitaxial relationship, we examined the TiO_2_ films grown on another (111)‐textured FCC metal layer. We selected Pd because of its FCC structure and lattice constant (0.389 nm), which is very close to that of Pt (0.392 nm). Figure 3j shows the θ–2θ XRD patterns of the TiO_2_ films grown on (111)‐textured Pd/amorphous SiO_2_/Si substrates. The θ–2θ XRD results verify that the Pd layer has a strong (111) preferential orientation, and TiO_2_ films with a rutile (110) preferential orientation were grown on this layer. Considering that TiO_2_ films grown on substrates without lattice matching to the metastable TiO_2_ phases predominantly crystallize into anatase (Figure S5, Supporting Information), this result supports the hypothesis that lattice matching between the FCC (111) surface and rutile (110) plane contributes to the stabilization of the rutile phase rather than the anatase phase. Additionally, rutile TiO_2_ is structurally more similar to orthorhombic TiO_2_‐II than to anatase, as both structures comprise Ti_3_O and Ti_2_O_2_Ti_2_ units.^[^ ^11^ ^]^ This structural similarity is believed to contribute to the preferential formation of rutile TiO_2_ in the studied systems.

Although the TiO_2_ films grown on polycrystalline Ir films with a preferential (111) orientation also crystallized into rutile (Figure S8, Supporting Information), the XRD patterns also show a Bragg peak corresponding to (110) IrO_2_. This is likely due to the oxidation of the polycrystalline Ir surface, which is rich in grain boundaries, during O_3_ exposure. Because IrO_2_ also has a rutile structure, the exact origin of rutile formation on polycrystalline Ir cannot be confirmed. A similar limitation arises for TiO_2_ films grown on polycrystalline Ni and Cu substrates, another FCC metal. The FCC metals were severely oxidized to NiO and CuO during TiO_2_ deposition, leading to the formation of anatase (Figure S9, Supporting Information).

**Figure 4:**
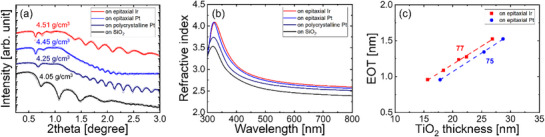
a) X‐ray reflectivity spectra and b) refractive indices of TiO_2_ films grown on epitaxial Pt, Ir (111) films, (111)‐textured polycrystalline Pt, and SiO_2_/Si. c) Variation in the EOT of TiO_2_ films grown on epitaxial Pt and Ir (111)/c‐cut sapphire as a function of TiO_2_ film thickness.

The physical and dielectric properties of metastable TiO_2_ phases, particularly orthorhombic TiO_2_‐II, have not been studied extensively. Therefore, we investigated the various properties of the TiO_2_‐II films. The densities of the TiO_2_‐II films grown on epitaxial Pt and Ir (111) films are 4.45 and 4.51 g cm^−3^, respectively, as estimated from the X‐ray reflectivity spectra in **Figure** **4**a. These values are higher than those of an anatase TiO_2_ film grown on SiO_2_ (4.05 g cm^−3^) and a rutile TiO_2_ film grown on polycrystalline Pt (4.25 g cm^−3^). These results demonstrate that TiO_2_‐II, stabilized as a high‐pressure phase, exhibits a significantly higher density than other common TiO_2_ polymorphs. TiO_2_‐II films grown on epitaxial Pt and Ir (111) substrates also show higher refractive indexes than those of anatase and rutile TiO_2_ films because the refractive index is closely related to the film density (Figure 4b).

The dielectric constants of the TiO_2_‐II phase estimated from the inverse slope of the equivalent oxide thickness (EOT) versus film thickness curves (Figure 4c) are ≈75 and 77 for films grown on epitaxial Pt (111) and Ir (111) substrates, respectively. These values are consistent with first‐principles predictions for the TiO_2_‐II phase.^[^ ^11^ ^]^ Furthermore, they are significantly higher than that of anatase TiO_2_, which has been reported to be ≈40, but slightly lower than the dielectric constant of rutile TiO_2_. Moreover, the dielectric constant remains unchanged under the operating voltage range of DRAM capacitors, highlighting the excellent phase stability of the TiO_2_‐II films.

The ALD‐grown TiO_2_‐II phase exhibits excellent stability as well. No phase transition was observed after two months of exposure to ambient conditions at room temperature or after annealing at 200 °C for 2 h in air (Figure S10, Supporting Information), demonstrating the excellent stability of the TiO_2_‐II films grown by ALD. These advantages suggest the potential as a dielectric material for DRAM capacitors.

## 3 Conclusion

The selective stabilization of metastable TiO_2_ polymorphs, particularly the orthorhombic TiO_2_‐II phase, was achieved by ALD on FCC metal substrates. The structural analysis revealed that the strong (111) orientation of the epitaxial Ir and Pt films promotes the growth of TiO_2_‐II with a dominant (200) orientation. In contrast, TiO_2_ films grown on polycrystalline FCC metals with a (111) texture preferentially crystallized into rutile, highlighting the critical role of in‐plane strain preservation in TiO_2_‐II stabilization. The TiO_2_‐II films exhibited higher densities, refractive indices, and dielectric constants than anatase TiO_2_, with dielectric constants (≈75–77) consistent with theoretical predictions. Our findings offer new insights into the strain‐mediated stabilization of high‐pressure TiO_2_ phases and provide a general strategy for the phase‐selective engineering of functional oxide thin films at low temperatures. This approach expands the applicability of metastable TiO_2_ phases in electronic and optical devices. In the future, employing more cost‐effective FCC metals as the substrate could further enhance the scalability and feasibility of this strategy.

## 4 Experimental Section

### TiO_2_ Film Growth

TiO_2_ films were grown in a commercial ALD chamber (Atomic Classic, CN‐1) at 330 °C. Trimethoxy(pentamethylcyclopentadienyl)titanium ((CpMe_5_)Ti(OMe)_3_; SK Trichem Co.) was used as the Ti precursor. The canister containing (CpMe_5_)Ti(OMe)_3_ was heated to 45 °C to inject a sufficient dose. O_3_ was supplied as the oxygen source at a concentration of 185 g Nm^−3^. A high concentration of O_3_ was generated using an inductive‐type O_3_ generator (Ozontech Co.) by flowing O_2_ at a rate of 500 sccm. A single ALD cycle consisted of Ti precursor injection (4 s), purging (15 s), O_3_ injection (3 s), and purging (10 s). The thickness of the film was controlled by varying the number of ALD cycles.

### Substrate Preparation

A layer of an FCC metal (Pt or Ir) was used as the substrate for TiO_2_ growth. To control the in‐plane orientation of the FCC metal layers, they were deposited on 100 nm‐thick SiO_2_/Si and c‐cut sapphire (iNexus, Inc.) substrates. Pt was deposited by DC sputtering on the SiO_2_ at room temperature and c‐cut sapphire substrates at 400 °C, whereas Pd was grown on SiO_2_ at room temperature via sputtering. Ir was deposited on c‐cut sapphire substrates at 300 °C using ALD. Tricarbonyl‐(1,2,3‐*η*)‐1,2,3‐tri(*tert*‐butyl)‐cyclopropenyl‐iridium (provided by TANAKA Precious Metals Technologies) was used as the Ir precursor, and O_2_ gas was used as the reactant at a flow rate of 100 sccm.

### Characterization

The film thickness was estimated using spectroscopic ellipsometry (MG‐1000, Nanoview). The crystal structure was examined by XRD (D8 DISCOVER, Bruker) in θ–2θ scan mode. EBSD analysis was performed using a field‐emission SEM (JSM‐7001F, JEOL) equipped with an EBSD system. The crystallographic orientation relationship between the TiO_2_ films and the substrate was examined using STEM and HRTEM (Tecnai G2 F20, FEI, and Titan 80–300, FEI). The dielectric constants of the TiO_2_ films were evaluated by fabricating metal–insulator–metal capacitors. The dielectric properties of the capacitors were measured using an Agilent 4294A impedance analyzer at 10 kHz.

## Conflict of Interest

The authors declare no conflict of interest.

## Supporting information



Supporting Information

## Data Availability

The data that support the findings of this study are available from the corresponding author upon reasonable request.
