# Supplementary material for: Strain‐Driven Selective Stabilization of Metastable TiO2 Phases
Source: Small. 2025 Sep 18;21(45):e05427. doi: 10.1002/smll.202505427 (PMC12614132; doi:10.1002/smll.202505427)
Supplement: Supplementary file 1 — Supporting Information [file SMLL-21-e05427-s001.pdf]

NANO · MICRO
small

Supporting Information

for *Small*, DOI 10.1002/smll.202505427

Strain-Driven Selective Stabilization of Metastable TiO_2 Phases

*Jihoon Jeon, Myungsu Jang, Gwang Min Park, Minseok Kim, Jongseo Kim, Seungwan Ye, Yongjoo Park, Seung-Hyub Baek, Jun-Yun Kang and Seong Keun Kim**

Supporting Information

Strain-Driven Selective Stabilization of Metastable TiO_2 Phases

*Jihoon Jeon, Myungsu Jang, Gwang Min Park, Minseok Kim, Jongseo Kim, Seungwan Ye, Yongjoo Park, Seung-Hyub Baek, Jun-Yun Kang, Seong Keun Kim**

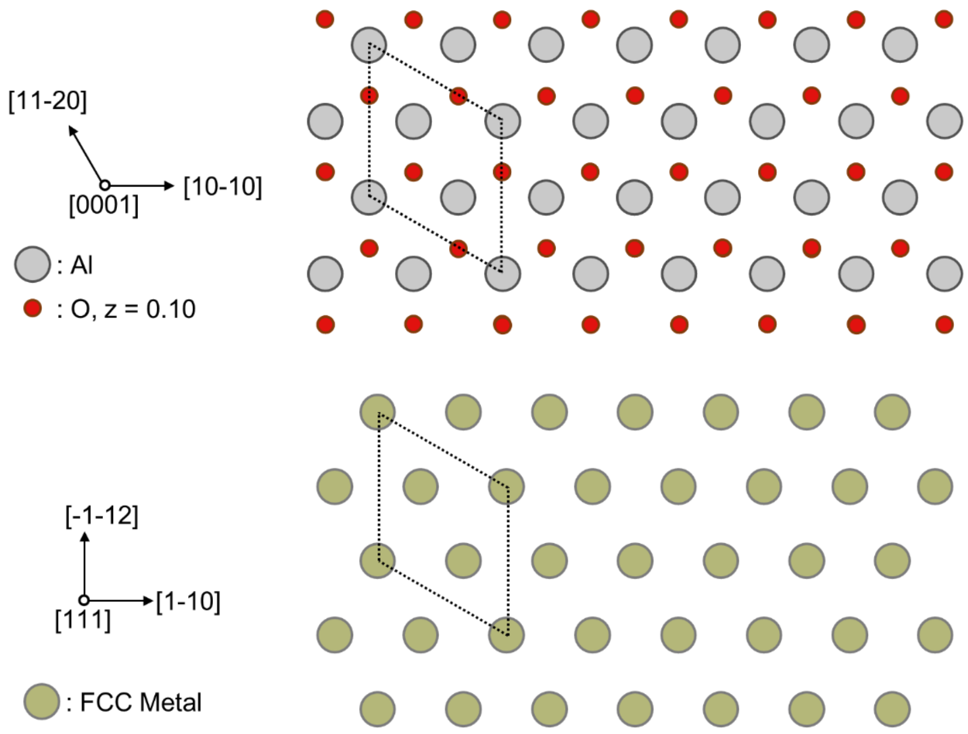

Figure S1. Schematics of the atomic arrangements of the (0006) plane of α-Al_2O_3 and the (111) plane of FCC metals.

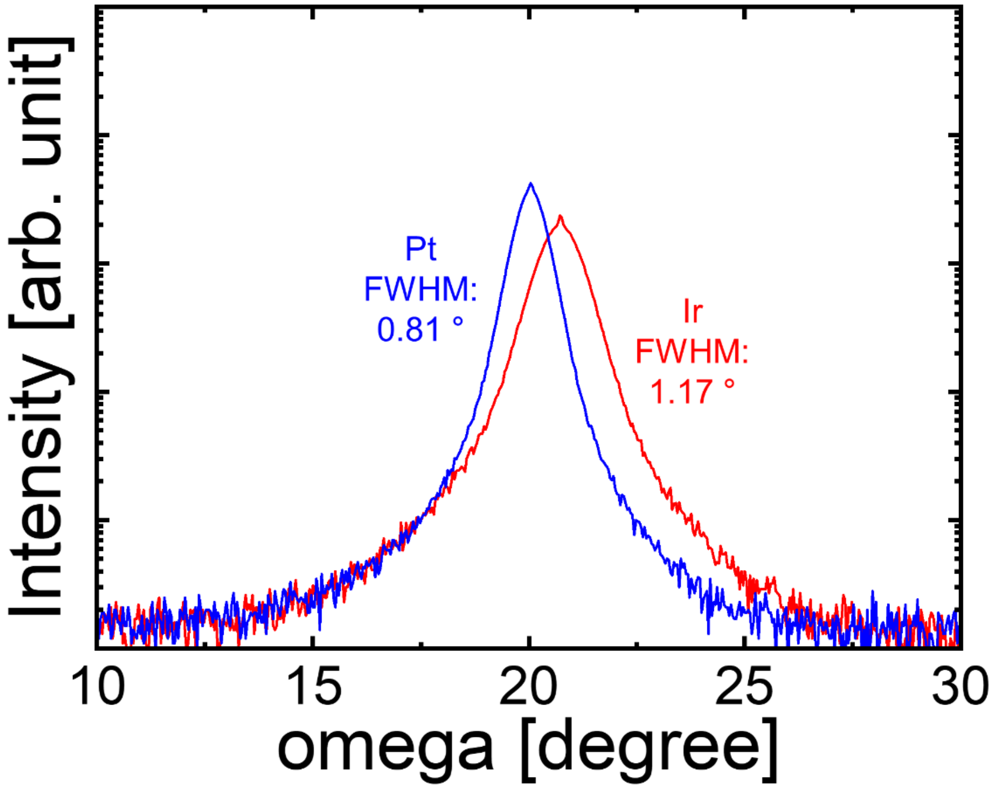

Figure S2. Rocking curves for the (111) reflections for Ir and Pt films grown on c-cut sapphire.

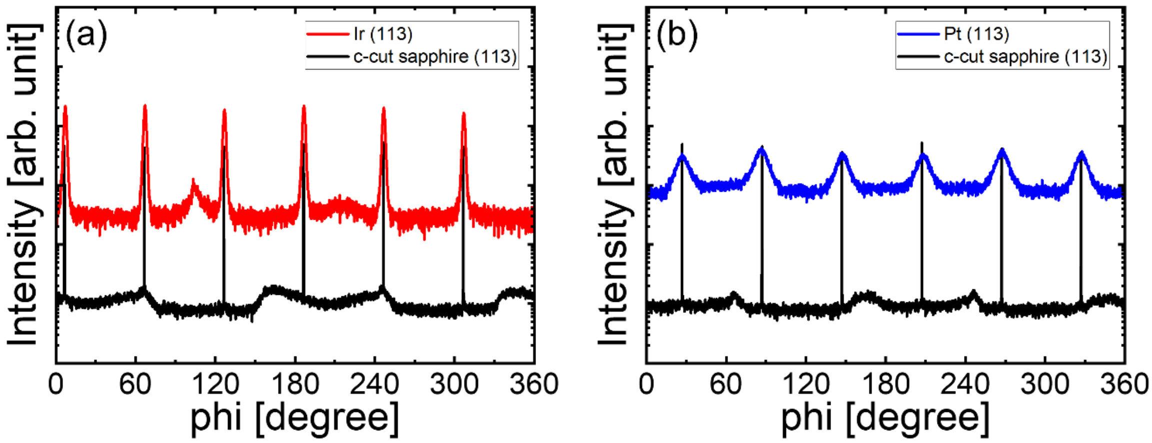

Figure S3. XRD φ scans of the Ir/α-Al_2O_3 and Pt/α-Al_2O_3.

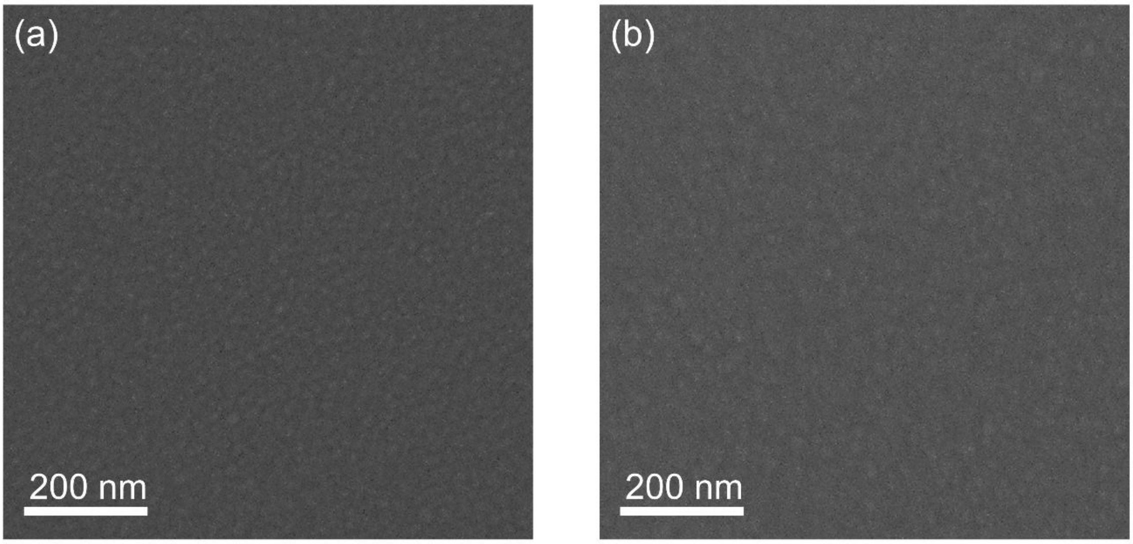

Figure S4. SEM images of TiO_2 films grown on epitaxial (a) Ir and (b) Pt layers grown on c-cut sapphire.

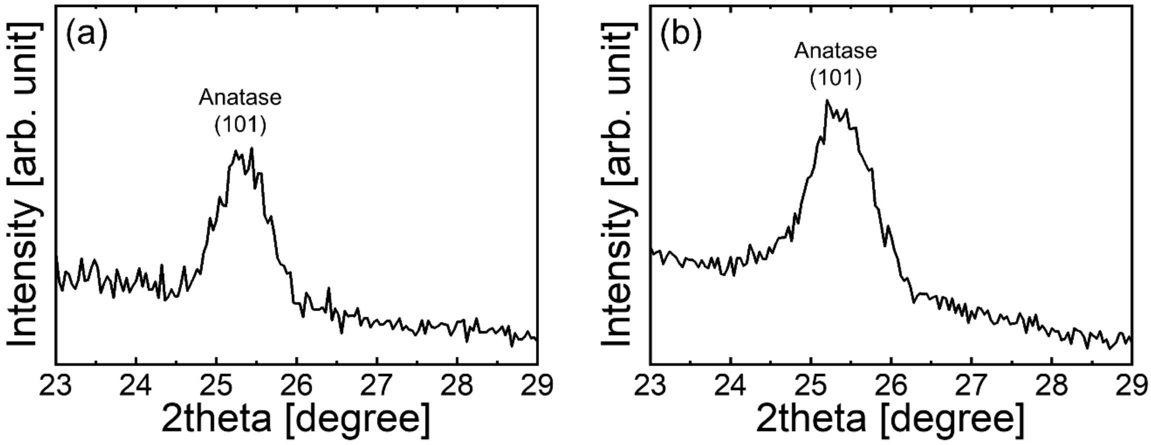

Figure S5. Grazing-incident XRD patterns of the TiO_2 films grown on (a) Si and (b) TiN.

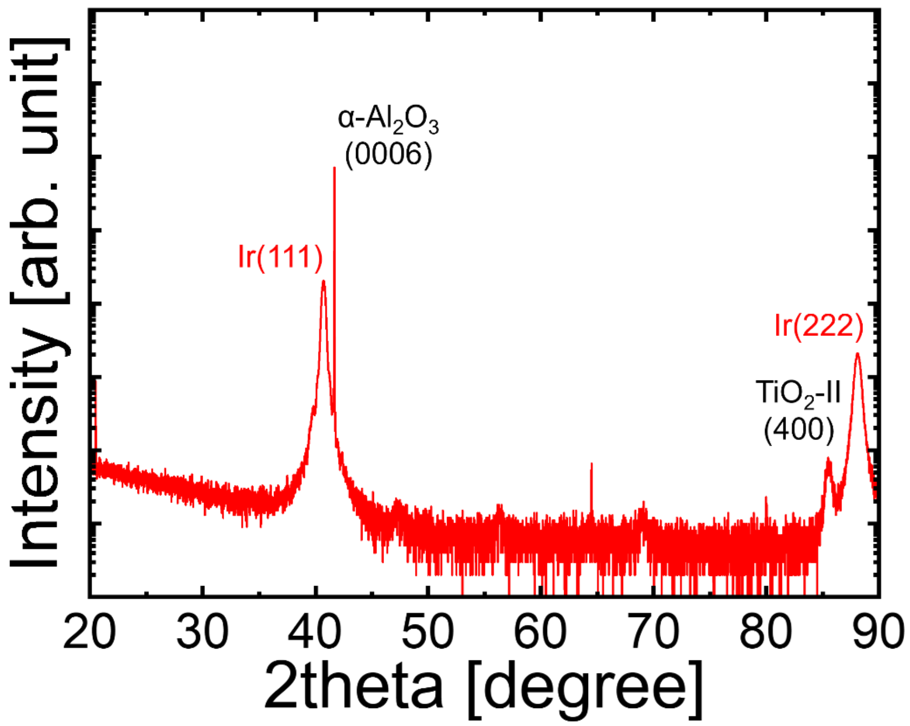

Figure S6. θ–2θ XRD patterns of 70 nm-thick TiO_2 films grown on Ir/c-cut sapphire.

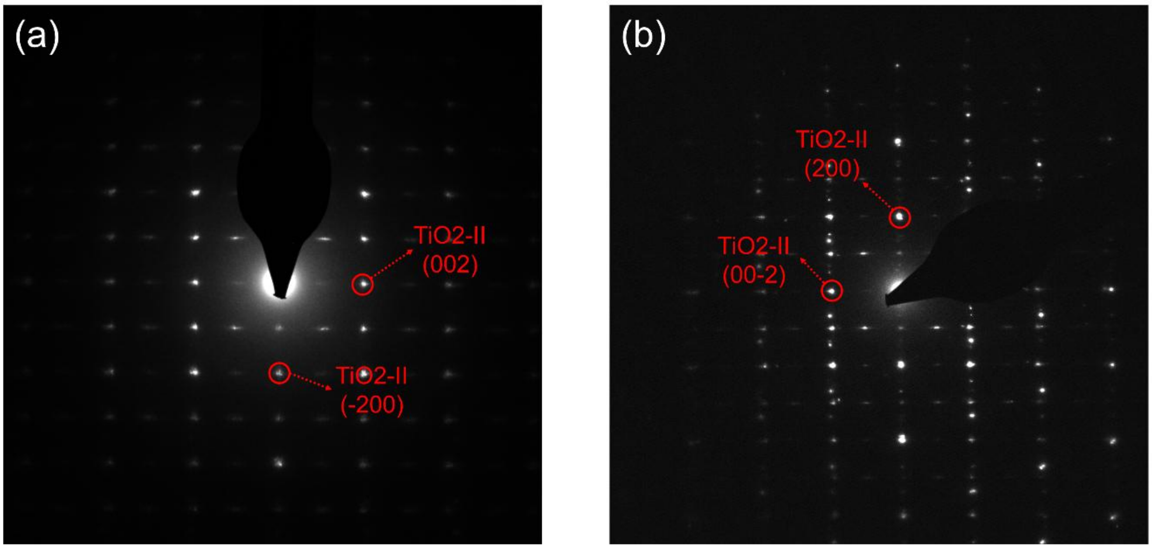

Figure S7. Selected area electron diffraction images of the TiO_2 films grown on epitaxial (a) Ir and (b) Pt layers grown on c-cut sapphire.

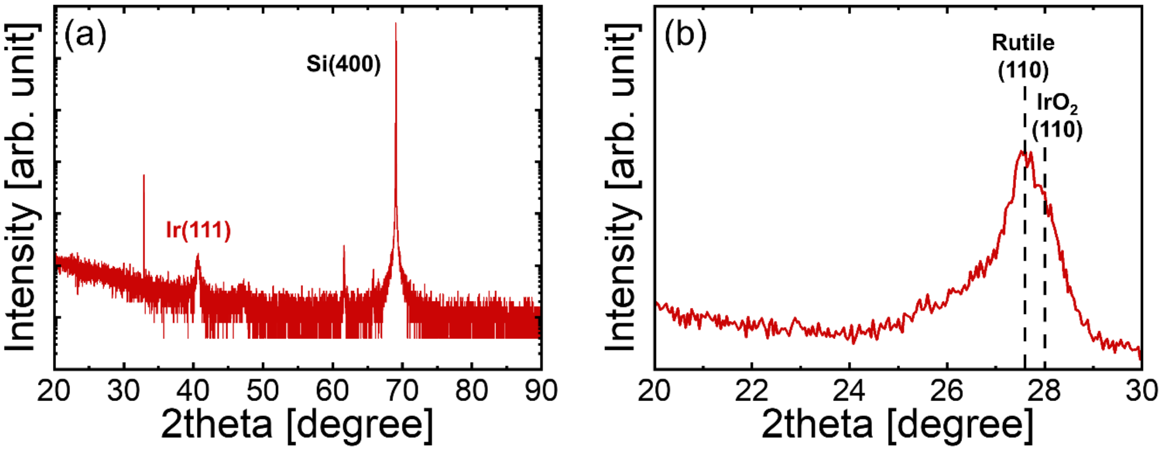

Figure S8. (a) θ–2θ XRD pattern of an Ir film grown on an amorphous SiO_2 substrate by ALD. (b) Grazing-incident XRD pattern of the TiO_2 film grown on the Ir film.

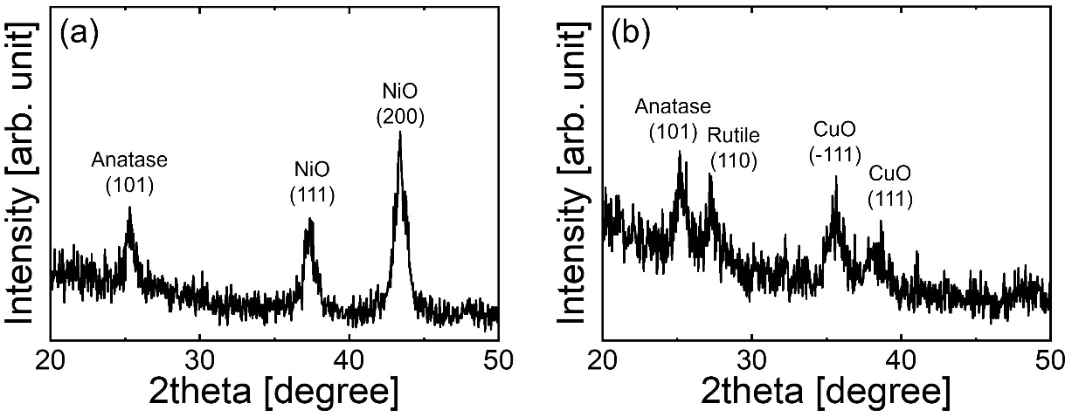

Figure S9. Grazing-incident XRD pattern of the TiO_2 films grown on (a) Ni and (b) Cu films.

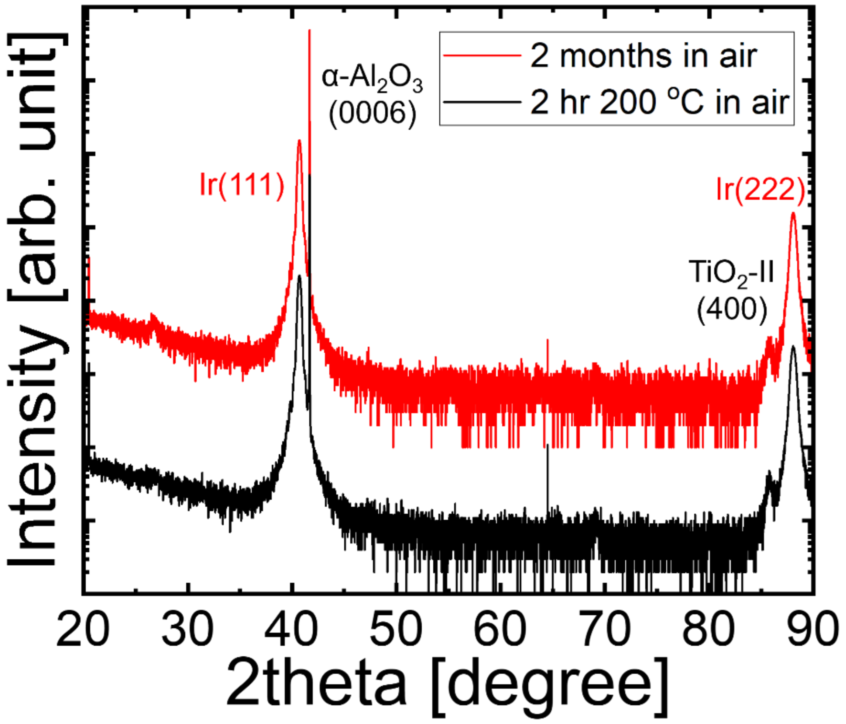

Figure S10. θ–2θ XRD patterns of TiO_2 films grown on Ir/c-cut sapphire after two months of exposure to ambient conditions at room temperature and after annealing at 200 °C for 2 hours in air.
